# Bile Acid Recognition by the *Clostridium difficile* Germinant Receptor, CspC, Is Important for Establishing Infection

**DOI:** 10.1371/journal.ppat.1003356

**Published:** 2013-05-09

**Authors:** Michael B. Francis, Charlotte A. Allen, Ritu Shrestha, Joseph A. Sorg

**Affiliations:** Department of Biology, Texas A&M University, College Station, Texas, United States of America; Boston Children's Hospital, United States of America

## Abstract

*Clostridium difficile* spores must germinate *in vivo* to become actively growing bacteria in order to produce the toxins that are necessary for disease. *C. difficile* spores germinate *in vitro* in response to certain bile acids and glycine. In other sporulating bacteria, proteins embedded within the inner membrane of the spore sense the presence of germinants and trigger the release of Ca^++^-dipicolinic acid (Ca^++^-DPA) from the spore core and subsequent hydrolysis of the spore cortex, a specialized peptidoglycan. Based upon homology searches of known germinant receptors from other spore-forming bacteria, *C. difficile* likely uses unique mechanisms to recognize germinants. Here, we identify the germination-specific protease, CspC, as the *C. difficile* bile acid germinant receptor and show that bile acid-mediated germination is important for establishing *C. difficile* disease in the hamster model of infection. These results highlight the importance of bile acids in triggering *in vivo* germination and provide the first description of a *C. difficile* spore germinant receptor. Blocking the interaction of bile acids with the *C. difficile* spore may represent an attractive target for novel therapeutics.

## Author Summary

*Clostridium difficile* infections (CDI) are steadily increasing in the United States and other countries. *C. difficile* spores are the infectious agent and often contaminate environmental surfaces. However, to initiate infection, *C. difficile* spores must germinate *in vivo* to actively growing bacteria. Certain bile acids and glycine are the most effective compounds that stimulate *C. difficile* spore germination. While the signals that stimulate germination by *C. difficile* spores are known, with what these compounds interact remained unknown. Here, we identified the germination-specific protease, CspC, as the bile acid germinant receptor. In *C. difficile*, CspC is not predicted to have catalytic activity. However, we find that mutations in *cspC* alter the specificity of germinant recognition or abrogate the ability of *C. difficile* spore to germinate in response to bile acids. Further, we show that bile acid recognition by *C. difficile* spores is important for establishing infection in an animal model of *C. difficile* disease. Our results suggest a unique mechanism for *C. difficile* spore germination through direct stimulation of cortex hydrolysis by a spore germinant. A detailed understanding of germinant recognition by *C. difficile* CspC may aid in the identification of germination-blocking compounds, which may have importance in hindering *C. difficile* colonization.

## Introduction

*Clostridium difficile* infections (CDI) are steadily increasing in the United States and other countries [1], [2]. The use of broad-spectrum antibiotics, often unrelated to CDI, leads to alteration of the colonic microbiota that normally provides resistance to *C. difficile* colonization [3]. In a host, *C. difficile* spores must germinate to form the actively growing, anaerobic bacteria that produce the two toxins that are necessary for disease (TcdA and TcdB) [4], [5], [6]. These two toxins are secreted by the bacterium where they then enter host epithelial cells by receptor-mediated endocytosis and, upon escape into the cytosol, glucosylate members of the Rho-family of GTPases [7]. The action of these toxins lead to symptoms normally associated with CDI (e.g. diarrhea) and release of *C. difficile* spores into the environment [8].

Metabolically dormant spores are formed by selected bacterial species in response to changes in environmental conditions, including nutrient availability [9]. During spore formation, the proteins required for germination are pre-packaged into the spore, priming the spore to germinate when conditions are appropriate [10]. In many spore-forming species, the interaction of the metabolically dormant spore with specific germination-inducing molecules (germinants) leads to the release of large amounts of Ca^++^-dipicolinic acid (DPA) from the dehydrated spore core in exchange for water. Subsequently, hydrolases embedded within the spore cortex, a specialized peptidoglycan, become activated and begin cortex hydrolysis. Once the core is rehydrated and the cortex is degraded, a vegetative cell begins to grow out from the germinated spore. This process is largely conserved among spore-forming bacteria, though the signals that initiate germination can vary. In *Bacillus subtilis*, L-alanine or a mixture of L-asparagine, glucose, fructose and potassium ions triggers germination, while spores of certain strains of *Clostridium perfringens* initiate germination in response to inorganic phosphate and sodium ions [11].

The proteins that respond to these signals, *ger* receptors, share homology among many spore-forming bacteria. However, based on homology searches, *C. difficile* is not among the spore-forming bacteria that have such canonical germinant receptors, suggesting that *C. difficile* responds to unique germinants or uses a novel mechanism for spore germination or both [12].

Approximately 30 years ago, Wilson and others showed that certain bile acids increased the frequency of *C. difficile* colony formation from environmental samples [13], [14], [15]. Bile acids are small amphipathic, cholesterol-based molecules that aid in the absorption of fats and cholesterol during digestion. Typically, the liver synthesizes two main bile acids, cholic acid (3α,7α,12α-trihydroxy-5β-cholanic acid) and chenodeoxycholic acid (3α,7α-dihydroxy-5β-cholanic acid), which are further modified with the addition of either a taurine or glycine amino acid [16]. Building on the work of Wilson and others, we demonstrated that all cholic acid derivatives can stimulate *C. difficile* colony formation from spores with approximately equal efficiency [17]. Further, we showed that exposure to the combination of taurocholic acid and glycine were required to initiate *C. difficile* spore germination [17]. Interestingly, chenodeoxycholic acid was unable to stimulate colony formation or the initiation of spore germination [17]. Subsequent studies identified chenodeoxycholic acid as a competitive inhibitor of cholic acid-mediated germination [18], [19]. While the chemical signals that promote the initiation of *C. difficile* spore germination are known, the proteins that respond to these germinants had not been identified.

Here, we applied a screen, previously used to identify loci involved in *B. subtilis* spore germination [20], to the identification of *C. difficile* germination-null phenotypes. Using a combination of traditional chemical mutagenesis and contemporary massively parallel DNA sequencing, we identified single nucleotide polymorphisms (SNPs) that give rise to *ger* phenotypes and characterized the resulting strains. We found that mutations in the *C. difficile cspC* gene can abrogate the initiation of *C. difficile* spore germination. Further, we identified a mutation in *cspC* that allows chenodeoxycholic acid to act as a spore germinant, instead of an inhibitor of germination. These results suggest that *C. difficile* CspC is the bile acid-sensing germinant receptor. The identification of the molecular target of bile acids on the *C. difficile* spore has allowed us to test, for the first time, the *in vivo* role of bile acid-mediated germination during *C. difficile* infection.

## Results

### Identifying germination-null phenotypes

**Figure 1:**
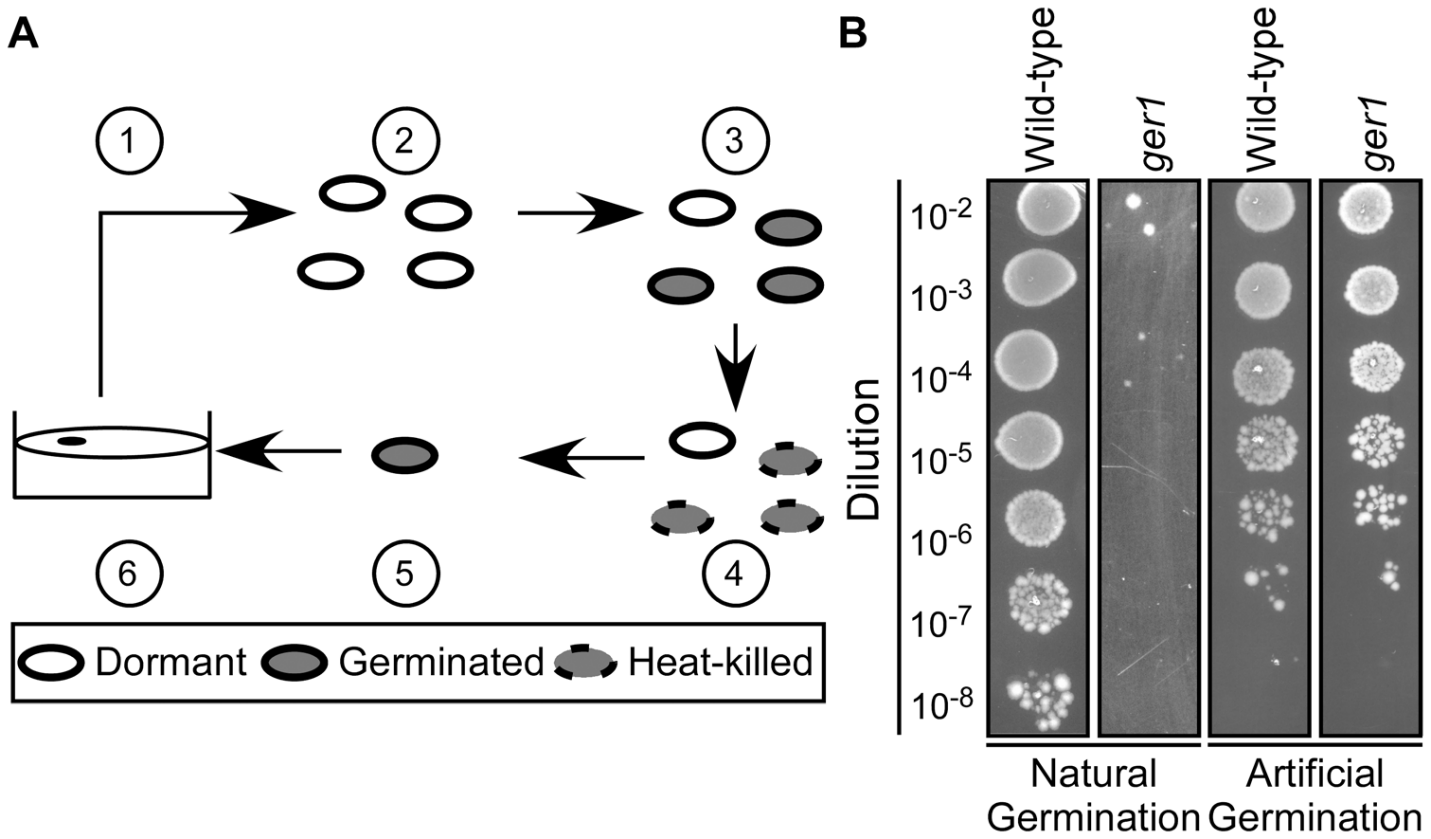
Isolation of *C.* *difficile* germination-null mutants. (A) Strategy to identify *C. difficile ger* phenotypes. Spores were generated (1) and purified (2). After purification, spores were germinated in BHIS medium supplemented with TA (3) and germinated spores heat-killed at 65°C (4). Spores that survived (4) were artificially germinated (5) before plating on BHIS medium (6). (B) *C. difficile* UK1 spores or *C. difficile ger1* spores were serially diluted and spotted on BHIS medium supplemented with 0.1% TA or germinated by thioglycollate/lysozyme and were serially diluted and spotted on BHIS medium.

Previously, we demonstrated that the cholic acid family of bile acids causes spores to initiate germination in rich medium [17]. To identify the *C. difficile* bile acid germinant receptor, we employed a strategy schematized in Figure 1A. We mutagenized *C. difficile* strain UK1 [19] using the DNA alkylating agent ethyl methanesulfonate (EMS). The EMS-mutagenized bacteria were allowed to recover during overnight incubation in fresh medium and spread on solid medium to allow efficient spore formation. Spores were purified and incubated overnight at 37°C in rich medium+10% w/v taurocholic acid [(TA); 185 mM] to germinate those spores that were still able to respond to TA as a germinant. The spore suspension (containing both germinated and non-germinated spores) was incubated at 65°C for 30 minutes to heat-kill the germinated spores; dormant, non-germinated spores are resistant to 65°C. The surviving spores were artificially germinated using thioglycollate and lysozyme [21] and then plated on rich medium to recover, as colonies, the artificially germinated spores. Mutants that failed to germinate under these conditions were enriched and 10 colonies, among thousands, were isolated and tested for the ability of their spores to germinate in response to TA.

Spores from all 10 isolates (*ger1–ger10*) were unable to form colonies on rich medium+TA, but did form colonies after artificial germination [21] (Figure 1B; only wild-type *C. difficile* UK1 and *ger 1* are shown). This suggests that the *ger* mutants are either blocked at the outgrowth stage of germination (inability to grow as a vegetative cell from the germinated spore) or blocked in the initiation of germination (inability to respond to TA as a germinant)

### Characterizing germination-null *C. difficile* strains

**Figure 2:**
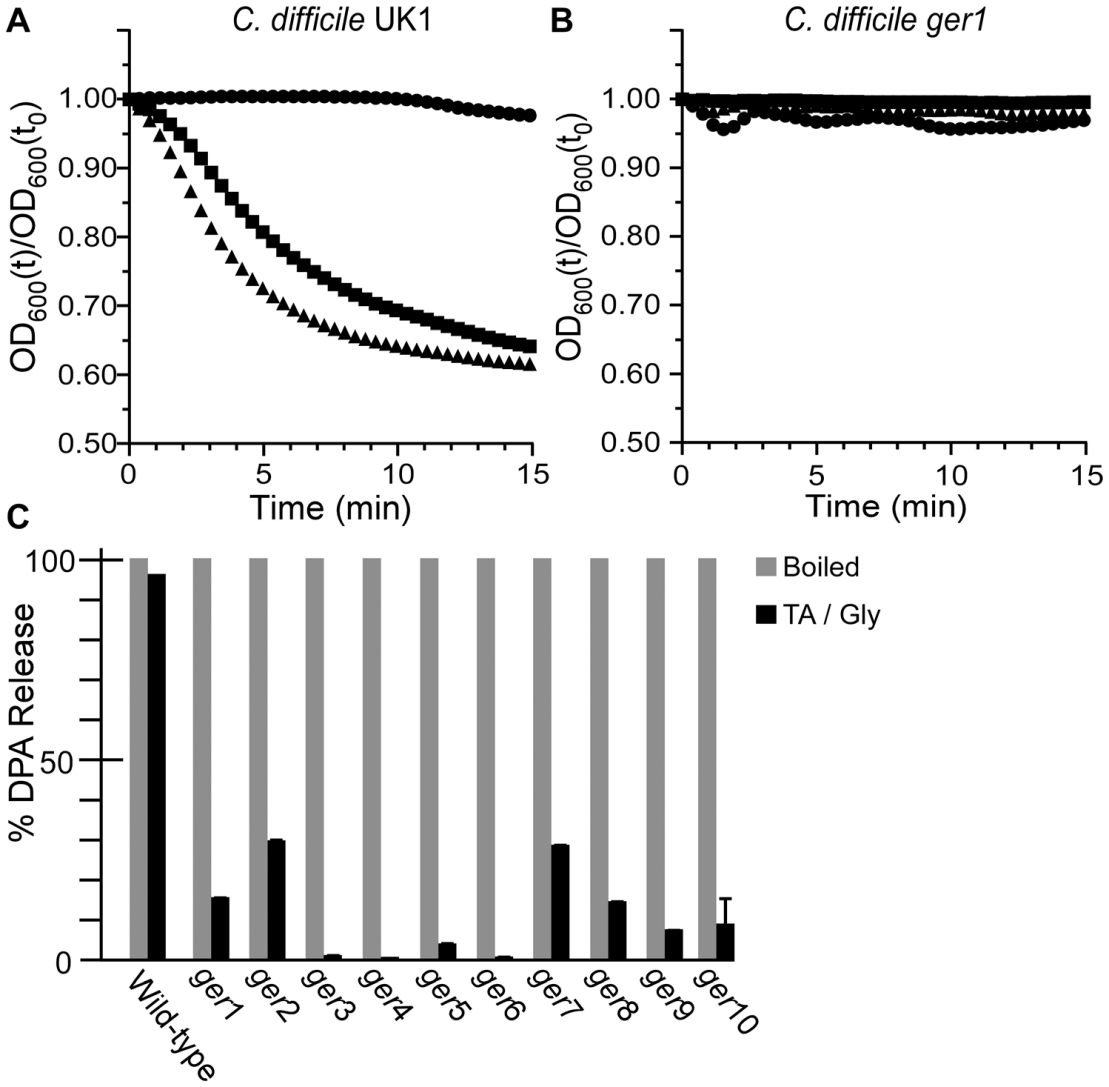
*C.* *difficile ger* isolates fail to initiate germination. Purified *C. difficile* UK1 spores (A) or *C. difficile ger1* spores (B) were suspended in BHIS medium (•) or BHIS medium supplemented with 5 mM TA (▪) or 50 mM TA (▴) and the initiation of germination was followed at A_600_. (C) DPA release from spores suspended in germination buffer supplemented with TA and glycine was analyzed at A_270_.

To determine at what stage the mutants are blocked, we analyzed the initiation of germination as measured by a decrease in A_600_ over time. Wild-type *C. difficile* UK1 initiated germination in the presence of 5 mM TA and 50 mM TA but not in the absence of TA (Figure 2A). However, spores derived from *C. difficile ger1* (Figure 2B) did not initiate germination even at the highest TA concentration used (50 mM). Also, while wild-type *C. difficile* released DPA in response to TA and glycine, *C. difficile ger1–ger10* spores were unable to release the majority of the stored DPA (Figure 2C); Ca^++^-DPA release from the spore core is one of the first measurable events in bacterial spore germination [10]. Together, these results suggest that the *C. difficile ger* isolates are defective in the earliest stages of spore germination and may be defective in recognizing TA as a germinant.

### Determining the locations of SNPs that give rise to *ger* phenotype

The locations of the SNP(s) that gave rise to the germination-null phenotypes were determined using Illumina sequencing technology (Table S1). The DNA sequence of the 10 *ger* isolates were compared to determine if all had mutations in the same locus or loci. All isolates had in common mutations in 7 loci, with 6 loci having conserved mutations among all isolates (Table 1). Interestingly, *C. difficile ger1–ger10* had several different mutations in the *cspBAC* locus (Table 2). In *Clostridium perfringens*, CspA, CspB and CspC are germination-specific proteases that cleave the spore cortex lytic enzyme, SleC, to the active form [22], [23], [24]. This allows precise control of the timing of cortex hydrolysis during germination. *C. perfringens* CspA, CspB and CspC, all members of the subtilisin-family of proteases, have identifiable catalytic triads, while, in *C. difficile*, only CspB has obvious catalytic residues. In wild-type *C. difficile*, *cspB* and *cspA* coding sequences have been fused. Further, the CspA and CspC catalytic triads appear to have been lost (Figure S1). Eight of the 10 mutant strains had mutations in *cspC* (Table 2), suggesting that, despite the apparent absence of catalytic activity, wild-type CspC may still have a role in *C. difficile* spore germination.

**Table 1:** Locations of the SNPs common to all 10 *C. difficile ger* isolates. Gene numbering is based upon *C. difficile* R20291 gene numbering.

Location	Mutation	Putative Function
Nt 129662	C→T	N/A
CD901	No aa change	Pseudogene
CD1323	Q138K	Putative rubrerythrin
CD1848	S308A	Putative peptidase
CD1913	K82fs	AraC-family transcriptional activator
CD2020	P211L	Putative histidine protein kinase
CspBAC	Various (see Table 2)	Germination-specific protease

Nt: nucleotide.

aa: amino acid.

fs: frame shift.

**Table 2:** Locations of *C. difficile cspBAC* mutations in the *ger* mutants.

Strain	Mutation(s)
*ger1*	CspC G171R
*ger2*	CspC G171R
*ger3*	CspC G483R
*ger4*	CspC V272G/S443N
*ger5*	CspBA Q632stp
*ger6*	CspC S488N
*ger7*	CspBA W359stp
*ger8*	CspC G171R
*ger9*	CspC G483R
*ger10*	CspC G276R

stp: stop codon.

### Defining the role of *C. difficile cspC* during spore germination

**Figure 3:**
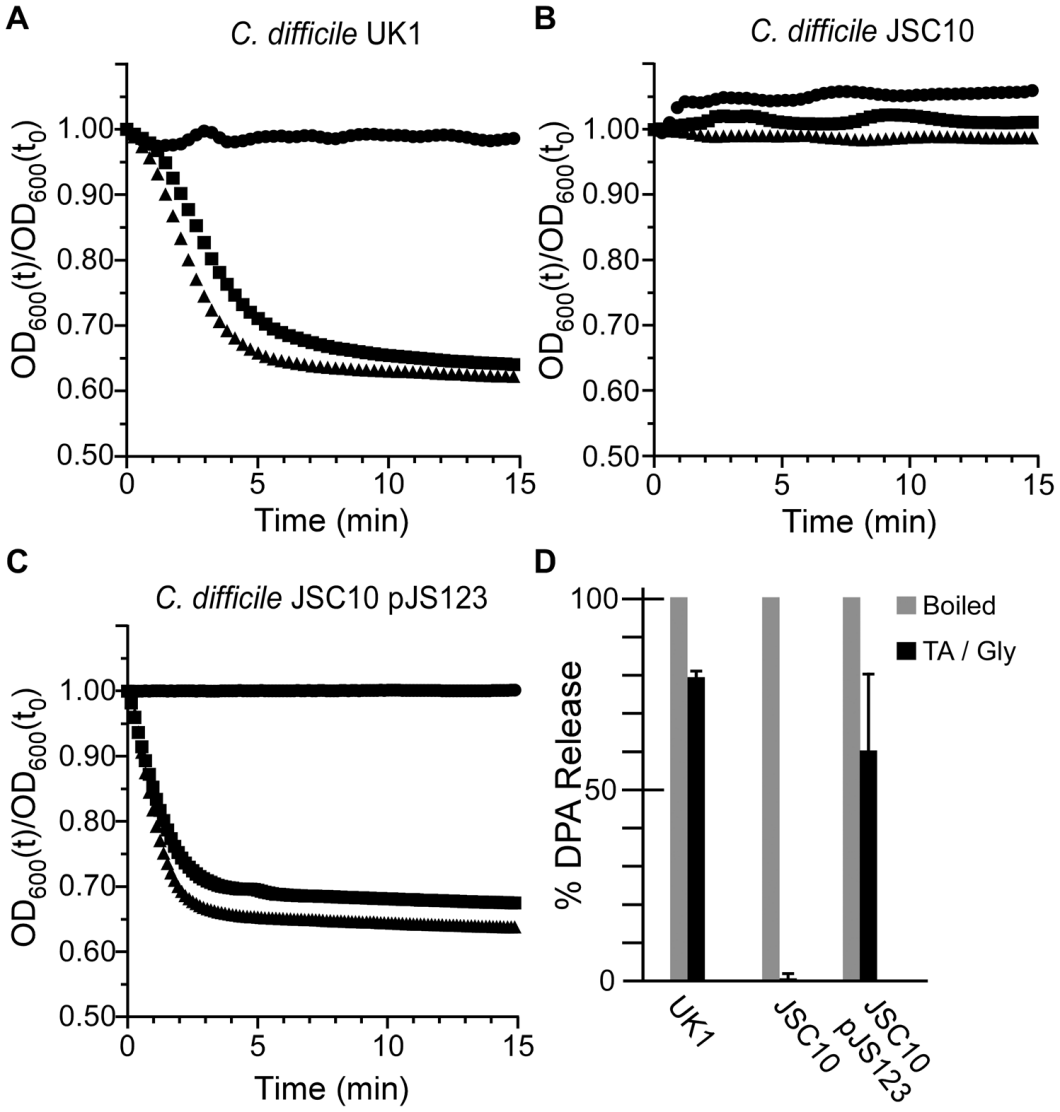
*C.* *difficile cspC* is essential for bile acid-mediated spore germination. Purified *C. difficile* UK1 spores (A) or *C. difficile* JSC10 (*cspC::ermB*) spores (B) or *C. difficile* JSC10 (*cspC::ermB*) pJS123 (p*cspBAC*) (C) were suspended in BHIS medium (•) or BHIS medium supplemented with 5 mM TA (▪) or 50 mM TA (▴) and the initiation of germination was followed at A_600_. (D) DPA release from spores suspended in germination buffer supplemented with TA and glycine was analyzed at A_270_.

To investigate the role of CspC in *C. difficile* spore germination, we generated a site-directed mutation using TargeTron technology [4], [25], [26], [27], [28]. The resulting strain, *C. difficile* JSC10 (*cspC::ermB*), is unable to initiate germination in response to TA (Figure 3B) unless provided with the *cspBAC* locus expressed *in trans* from a plasmid (Figure 3C); wild-type *C. difficile* UK1 initiates germination in response to TA (Figure 3A). Interestingly, when *C. difficile* JSC10 was complemented with the *cspBAC* locus, spores generated from this strain appear to germinate more rapidly than do *C. difficile* UK1 spores. Further work will be needed to characterize the germination rates of these spores. When analyzed for DPA release, wild-type *C. difficile* UK1 and *C. difficile* JSC10 (pJS123) released DPA while *C. difficile* JSC10 was unable to release DPA (Figure 3D).

### Mutations in *cspC* can alter germinant specificity

**Figure 4:**
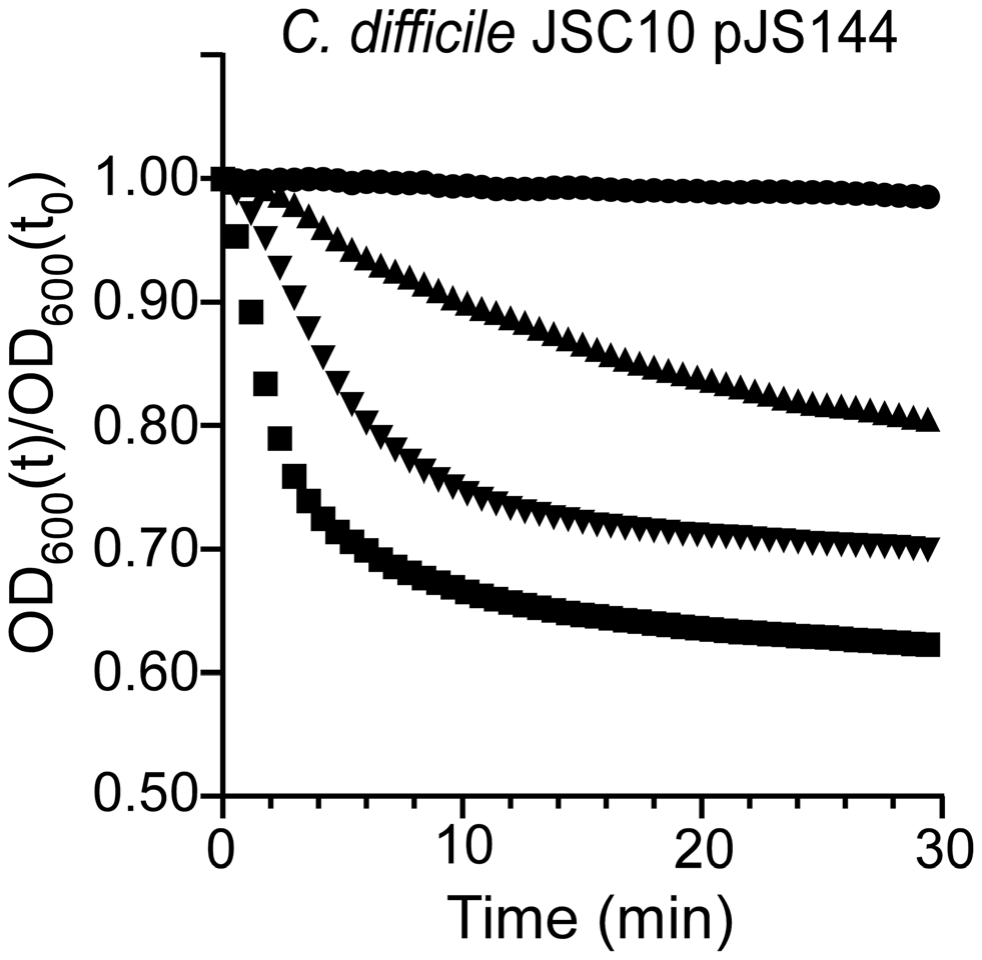
Mutations in *C.* *difficile cspC* can alter germination specificity. Purified *C. difficile* JSC10 (*cspC::ermB*) pJS144 (p*cspBAC_G457R_*) spores were suspended in BHIS medium (•) or BHIS medium supplemented with 1 mM chenodeoxycholic acid (▴) or 5 mM chenodeoxycholic acid (▾) or 10 mM TA (▪) and the initiation of germination was followed at A_600_.

It was previously reported that a mutation in *sleC* prevents *C. difficile* spore germination [27]. Thus, mutations that affect germination do not necessarily indicate that the gene in which the mutation lies normally codes for a germinant receptor. To test the hypothesis that *C. difficile* CspC is a *bona fide* germinant receptor, we again mutagenized *C. difficile* UK1 and allowed the mutagenized bacteria to form spores. The purified spores were plated on BHIS medium supplemented with 0.5 mM chenodeoxycholic acid. We looked for colony formation after 48 hours of incubation at 37°C. Chenodeoxycholic acid is a competitive inhibitor of cholic acid-mediated germination for *C. difficile* UK1 [18], [19] and other *C. difficile* strains [29]. Thus, in order to form colonies, these spores must have acquired an altered germinant specificity. Colonies were isolated and the phenotype confirmed as described above. We sequenced *cspC* from these newly generated strains and identified a single mutation, G457R. When the *cspC_G457R_* allele was used to complement *C. difficile* JSC10, we observed that this strain germinated in response to either TA or chenodeoxycholic acid (Figure 4). These results suggest that *C. difficile* CspC is a receptor for bile acid germinants.

### Bile acid-mediated germination is important for *C. difficile* infection in hamsters

**Figure 5:**
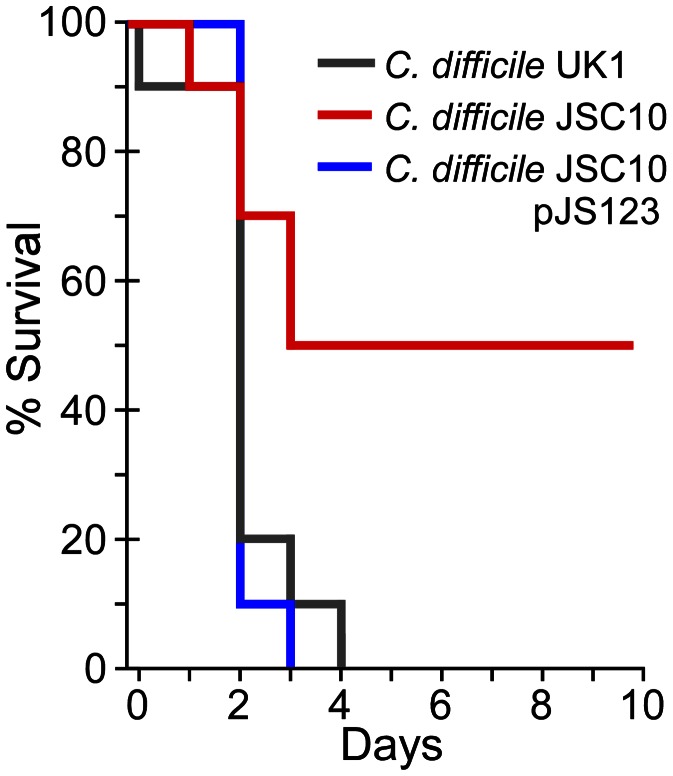
Bile acid-mediated germination is required for virulence. Kaplan-Meier survival curve of clindamycin-treated Syrian hamsters inoculated with 1,000 spores of *C. difficile* UK1 or *C. difficile* JSC10 (*cspC::ermB*) or *C. difficile* JSC10 (*cspC::ermB*) pJS123 (p*cspBAC*). Animals showing signs of *C. difficile* infection (wet tail, poor fur coat, lethargy) were euthanized.

The *in vivo* signals that trigger *C. difficile* spore germination are unknown, though bile acids are obvious candidates [30]. To test whether bile acid-mediated germination is required for *C. difficile* infection, Syrian hamsters were treated with clindamycin to induce sensitivity to *C. difficile* colonization and infection; the Syrian hamster has been used for approximately 30 years to assess *C. difficile* virulence and recapitulates the most severe form of human *C. difficile* infection, pseudomembranous colitis [31]. Hamsters were gavaged with 1,000 *C. difficile* UK1 spores or *C. difficile* JSC10 spores or *C. difficile* JSC10 (pJS123) spores and monitored for signs of CDI. Animals infected with either *C. difficile* UK1 or *C. difficile* JSC10 (pJS123) rapidly succumbed to disease. However, *C. difficile* JSC10 was unable to cause fulminant CDI and exhibited reduced virulence (Chi-squared: p-value<0.02) (Figure 5). These results show that bile acid-mediated germination is important for *C. difficile* disease and suggest that inhibiting *C. difficile* spore germination may have therapeutic potential.

## Discussion

Classically, germinant receptors are embedded within the inner membrane of bacterial spores [10], [32]. Germinants must pass through layers of coat proteins, an outer membrane, the cortex and germ cell wall before interacting with their respective receptors. Upon interaction, Ca^++^-DPA is released from the spore core in exchange for water. This exchange is essential to rehydrate the core and allow metabolism to begin. In some bacteria, the release of Ca^++^-DPA triggers the activation of cortex hydrolases allowing a vegetative bacterium to grow from the germinating spore [33]. In *C. perfringens* the germination-specific proteases cleave the cortex hydrolase, SleC, to an active form [23], [24]. The signals that stimulate this proteolysis in *C. perfringens* are not known. In *C. perfringens*, CspA, CspB and CspC are all members of the subtilisin family of serine proteases and have complete catalytic triads, suggesting that any one of these proteins can activate SleC-mediated cortex hydrolysis. In *C. difficile*, the *cspB* and *cspA* coding sequences have been fused [34]. Only CspB contains a complete catalytic triad while CspA and CspC have lost their catalytic residues. Based on sequence analysis, one would predict that only CspB would have an active role in stimulating *C. difficile* cortex hydrolysis. Indeed, a recent study by Adams and colleagues has shown that CspBA undergoes autoprocessing to generate CspB, which can cleave the cortex hydrolase pro-SleC to an active form [34]. We have provided evidence that *C. difficile* CspC plays an active and essential role during germination by functioning as the bile acid germinant receptor.

In *C. difficile* CspC, two of the three catalytic residues have been lost, T170 (conserved H198 in *C. perfringens* CspC) and G485 (conserved S517 in *C. perfringens* CspC) (Figure S1 – red). Interestingly, several the SNPs identified in the germination-null screen lie near T170 or G485 (Figure S1 – green). When we screened for *C. difficile* mutants that germinated in response to an inhibitor of germination (chenodeoxycholic acid), we identified G457R (Figure S1 – yellow). This residue is approximately 30 amino acids removed from G485. G457R, being a fairly drastic substitution, may modify the bile acid binding pocket to allow for a less-stringent recognition of germination-inducing bile acids. The 12α-hydroxyl group that differentiates between cholic acid and chenodeoxycholic acid protrudes from the molecule. This hydroxyl, in wild-type CspC, may penetrate the hypothetical binding pocket, resulting in a conformational change that is transmitted to *C. difficile* CspB [34]. CspB would then cleave SleC, initiating cortex hydrolysis [34]. Two of the identified SNPs in the germination-null screen were nonsense mutations in *cspBA* (Q632stop and W359stop). In the CspBA hybrid protein, Q632 is located in CspA while W359 is in CspB. The generation of a premature stop codon in *cspB* would result in a truncated protein with an incomplete catalytic triad [34]. The precise role of CspA in *C. difficile* spore germination is unknown, though our data suggest that CspA may be important.

Our data indicate that host-derived bile acids mediate *C. difficile* spore germination and that recognition of bile acids is required for infection in the hamster model to be maximally effective. Still, 50% of the animals succumbed to disease when infected with the *cspC* mutant, suggesting (i) that enough spores spontaneously germinated in the GI tract of the animal to cause disease, or (ii) that other, as yet unidentified, host signals can stimulate spore germination. Lysozyme is able to induce germination of *C. difficile* spores *in vitro* [21]. However, recent evidence has suggested that lysozyme at physiological levels may not be able to stimulate *C. difficile* spore germination [35] and we observe most efficient lysozyme-mediated spore germination after spore coat removal. Further study will be needed to determine if other signals provided by the host can stimulate *C. difficile* spore germination.

Previously, we identified inhibitors of *C. difficile* spore germination that had increased potency when compared to chenodeoxycholic acid [19]. It is not yet known whether these germination inhibitors have therapeutic importance but a recent study by Howerton and coworkers has shown dosing antibiotic-treated mice with an inhibitor of germination can reduce disease severity [36]. The identification of the molecular target of bile acids in the *C. difficile* spore may allow even more potent inhibitors to be rationally designed. Further, these inhibitors may aid in the identification in the bile acid-binding pocket in CspC by providing high-affinity interaction, instead of the relatively low affinity (in the mM range) for taurocholic acid [19], [37].

The relative affinities of bile acids for the *C. difficile* spore were determined using kinetics of germination [18], [19], [37], [38], [39]. While these studies were important milestones in determining which bile acids affect *C. difficile* germination and what features are important for triggering or inhibiting germination, the precise interaction of the bile acid with the germinant receptor has not been analyzed. Also, it has been proposed that the bile acid germinant receptor binds taurocholic acid cooperatively [37]. The identification of the bile acid germinant receptor now permits testing these interactions.

Stimulation of cortex hydrolysis may not be sufficient to fully activate *C. difficile* spore germination. *C. difficile* spores suspended in buffered taurocholic acid alone do not initiate spore germination unless a co-germinant, glycine, is added [17]. This suggests that a second, glycine-sensing receptor is required to trigger germination and the return to vegetative growth. We hypothesize that this other receptor may be localized to the inner membrane to aid in the release of Ca^++^-DPA from the spore core during germination.

## Materials and Methods

### Ethics statement

All animal procedures were performed with prior approval from the Texas A&M Institutional Animal Care and Use Committee. Animals showing signs of disease were euthanized by CO_2_ asphyxia followed by thoracotomy as a secondary means of death, in accordance with Panel on Euthanasia of the American Veterinary Medical Association. Texas A&M University's approval of Animal Use Protocols is based upon the United States Government's Principles for the Utilization and Care of Vertebrate Animals Used in Testing, Research and Training and complies with all applicable portions of the Animal Welfare Act, the Public Health Service Policy for the Humane Care and Use of Laboratory Animals, and all other federal, state, and local laws which impact the care and use of animals.

### Bacterial strains and growth conditions

*C. difficile* UK1 [19] (Table 3) was grown in a Model B, Coy Laboratory Chamber at 37°C under anaerobic conditions (85% nitrogen, 10% hydrogen, 5% carbon dioxide) in BHIS medium (Brain Heart Infusion supplemented with 5 g/L yeast extract and 0.1% L-cysteine). Antibiotics were added as needed (20 µg/ml thiamphenicol, 10 µg/ml lincomycin, 5 µg/ml rifampin). *E. coli* DH5α [40] was routinely grown at 37°C in LB medium. Antibiotics were added as needed (50 µg/ml kanamycin or 20 µg/ml chloramphenicol). *Bacillus subtilis* was grown at 37°C in LB medium and antibiotics were added as needed (2.5 µg/ml chloramphenicol, 5 µg/ml tetracycline).

**Table 3:** Strains and plasmids used in this study.

Strain	Description/Phenotype	Reference
*E. coli* DH5α	F^−^ *endA1 glnV44 thi-1 recA1 relA1 gyrA96 deoR nupG* Φ80d*lacZ*ΔM15 Δ(*lacZYA-argF*)U169, *hsdR17*(r_K_^−^ m_K_^+^), λ–	[40]
*B. subtilis* Bs49	Tn*916* donor strain, Tet^R^	[48]
*C. difficile* UK1	Wild type, PCR ribotype 027	[19]
*C. difficile* JSC10	*cspC* TargeTron mutant, germination null	This study
**Plasmids**		
pMTL84151	*E. coli-C. difficile* shuttle vector (pCD6 ColE1 *traJ* Cm^R^)	[44]
pBL100	TargeTron vector	[43]
pJS107	Tn*916 oriT* in pBL100	This study
pJS116	*B. subtilis-C. difficile* shuttle vector (pCD6 ColE1 Tn*916 oriT* Cm^R^)	This study
pJS123	*cspBAC* locus cloned in pJS116, complements *cspC* mutation	This study
pJS130	*cspC*-targeted TargeTron in pJS107	This study
pJS144	*cspBAC_G457R_* locus cloned in pJS116	This study

### EMS mutagenesis

One overnight culture of *C. difficile* UK1 was diluted 1∶100 in 5 ml fresh medium and grown to OD_600_ = 0.5 before adding ethyl methanesulfonate (EMS) to 1% final concentration. The culture was incubated for 3 hours, washed in BHIS medium and recovered overnight in 40-ml BHIS. A sample was taken to score mutation frequency on rifampin-containing BHIS agar medium (Table S2) and 50-µL samples were spread on 20 BHIS plates to allow spore formation of the mutagenized bacteria. Plates were incubated for 4 days before spores were harvested and purified as described previously [19]. Purified spores were suspended in 40 ml BHIS+10% w/v taurocholic acid (TA) and incubated overnight at 37°C to germinate those spores that recognized TA as a germinant. Spores were collected and heated to 65°C for 1 hour to inactivate germinated spores (dormant spores are resistant to 65°C). To germinate the remaining dormant spores, spores were again collected and treated with 250 mM thioglycollate for 30 min at 50°C followed by incubation with 4 mg/ml lysozyme for 15 min at 37°C [21] and 25-µL aliquots were spread on BHIS agar plates to allow spore formation. To enrich for germination null phenotypes, spores were again collected and germinated as described above.

To select for mutations that change the affinity of the germinant receptor from TA to chenodeoxycholic acid, *C. difficile* UK1 was mutated as described above with the following modification. Purified spores generated from mutated bacteria were spread on BHIS medium supplemented with 0.5 mM chenodeoxycholic acid. Colonies from spores that germinated on this medium were purified and the germination phenotype of their spores was confirmed using standard germination techniques (below).

### Illumina sequencing

High-quality, high-molecular weight genomic DNA was extracted, as described previously [41], [42], and submitted to Tufts University School of Medicine Genomics Core facility for Paired-End 50 Illumina re-sequencing. The samples were sonicated in a 4°C water bath with a Branson sonicator. Illumina libraries were then prepared using the Illumina TruSeq genomic DNA kit and tagged with individual Illumina barcodes. Final libraries were checked on said advanced analytical device, and then diluted to 10 nM prior to being loaded on a lane of an Illumina HiSeq2000. Illumina single-end sequencing was carried out for 50 cycles. The resulting sequence data in fastq format was aligned against the *C. difficile* R20291 genome using CLC Genomics Workbench, and SNPs were called at any position where more than 66% of the reads had an alternate base from the reference

### Molecular biology

The Tn*916 oriT* from *Bacillus subtilis* Bs49 was amplified using oligonucleotides 5′Tn916SLIC and 3′Tn916SLIC (Table 4) and introduced into the BstAPI restriction site of pBL100 [43] using Sequence and Ligation Independent Cloning (SLIC), generating pJS107. The pJS107 plasmid was used as a TargeTron vector to introduce mutations in *to C. difficile*. The group II intron insertion sites for *C. difficile cspC* were identified using an algorithm that can be found at http://dna.med.monash.edu.au/~torsten/intron_site_finder/. The intron fragment was generated as described previously using oligonucleotides cspC (115) EBS2, cspC (115) IBS, cspC (115) EBS1 and EBSU, cloned into pCR2.1-TOPO and then sub-cloned at the HindIII and BsrGI sites of pJS107, yielding pJS130. The *B. subtilis – C. difficile* shuttle vector, pJS116, was generated through the introduction of the Tn*916 oriT* into the ApaI restriction site of the *E. coli – C. difficile* shuttle vector, pMTL84151 [44], using oligonucleotides 5′Tn916ApaI and 3′Tn916ApaI which amplify the Tn*916 oriT*. The *C. difficile cspBAC* loci were amplified with Phusion polymerase using 5′cspBA_CXbaI and 3′cspBA_CXhoI oligonucleotides and cloned into the *B. subtilis – C. difficile* shuttle vector, pJS116. The nucleotide sequences for all constructs were confirmed before use.

**Table 4:** Oligonucleotides used in this study.

5′Tn916SLIC	5′ – GCAGATTGTACTGAGAGTGCACCATTAA CAT CTTCTA TTTTTCCCAAATCC – 3′
3′Tn916SLIC	5′ – ATCTGTGCGGTATTTCACACCGCATCTAAAGGGAATGTAGATAAATTATTAGGTAATC – 3′
cspC (115) EBS2	5′ – TGAACGCAAGTTTCTAATTTCGGTTAAAATCCGATAGAGGAAAGTGTCT – 3′
cspC (115) IBS	5′ – AAAAAAGCTTATAATTATCCTTAATTTTCAATAATGTGCGCCCAGATAGGGTG – 3′
cspC (115) EBS1	5′ – CAGATTGTACAAATGTGGTGATAACAGATAAGTCAATAATATTAACTTACCTTTCTTTGT – 3′
EBSU	5′ – CGAAATTAGAAACTTGCGTTCAGTAAAC – 3′
5′Tn916ApaI	5′ – AAGGGCCCTAACATCTTCTATTTTTCCCAAATCC – 3′
3′Tn916ApaI	5′ – AAGGGCCCCTAAAGGGAATGTAGATAAATTATTAGGTAATC – 3′
5′cspBA_CXbaI	5′ – AATCTAGAAAAACTATAAAGTTATAATTGTTGG – 3′
3′cspBA_CXhoI	5′ – AACTCGAGCTATAGAGTATTTGCTATCTGTTGA – 3′

### Conjugation and mutant selection

*B. subtilis* BS49 was used as a donor for conjugation with *C. difficile*. Plasmids were introduced into *B. subtilis* BS49 using standard techniques. Conjugation experiments were carried out as described previously [28]. *C. difficile* transconjugants were screened for the presence of Tn*916* using tetracycline resistance. Thiamphenicol-resistant, tetracycline-sensitive (plasmid-containing, transposon negative) transconjugants were selected for further use. Potential TargeTron mutants were generated by screening lincomycin-resistant *C. difficile* for the insertion of the intron into *C. difficile cspC* using primers specific for full-length *C. difficile cspC*, the 5′ intron insertion site and the 3′ intron insertion site and a positive clone was identified, *C. difficile* JSC10.

### Spore germination and DPA release

Spores were purified from BHIS agar medium as described previously [19] with the following modification. Spores from antibiotic-resistant strains (i.e. plasmid-containing or mutant strains) were generated on SMC medium [45] supplemented with appropriate antibiotics and purified as described previously. The initiation of spore germination was analyzed in a Lambda 25 Perkin Elmer spectrophotometer at A_600_ every 18 seconds, as described previously [17], [18], [19]. DPA release was measured by incubating purified spores at 37°C in germination salts (0.3 mM (NH_4_)_2_SO_4_, 6.6 mM KH_2_PO_4_, 15 mM NaCl, 59.5 mM NaHCO_3_ and 35.2 mM Na_2_HPO_4_) supplemented with 10% TA and 1 mM glycine for 1 hour. Equal aliquots were incubated at 100°C as a measure of 100% DPA release (positive control) or incubated at 37°C in germination salts without TA addition (negative control). Spores were sedimented and the supernatant was analyzed at A_270_ to measure the released DPA [46].

### Virulence studies

Female Syrian golden hamsters, 80 g–120 g, were housed individually in cages and had *ad libitum* access to food and water for the duration of the experiment. To induce susceptibility to *C. difficile* infection, hamsters were gavaged with 30 mg/kg clindamycin [31], [47]. After 5 days, hamsters were gavaged with 1,000 spores of *C. difficile* UK1 or *C. difficile* JSC10 or *C. difficile* JSC10 pJS123, 10 animals per strain, and monitored for signs of disease (lethargy, poor fur coat and wet tail). Hamsters showing signs of disease were euthanized by CO_2_ asphyxia followed by thoracotomy as a secondary means of death in accordance with Panel on Euthanasia of the American Veterinary Medical Association. Fecal samples were collected daily and cecum samples were collected on those hamsters requiring euthanasia. All animal studies were performed with prior approval from the Texas A&M University Institutional Animal Care and Use Committee.

### Statistical analyses

Experiments were performed in triplicate and, where indicated, error bars represent 1 standard deviation from the mean. A representative sample for the initiation of germination experiments at A_600_ is shown, error bars obscure the data. The data varied by <5%. Statistical significance of DPA release was performed using the Student's T-test. Differences in hamster survival between those infected with *C. difficile* JSC10 and either *C. difficile* UK1 or *C. difficile* JSC10 pJS123 were analyzed using the Log-rank test (GraphPad Prism).

## Supporting Information

Figure S1 **Sequence alignment between** ***C. difficile*** **CspC and** ***C. perfringens*** **CspC.** *C. difficile* CspC and *C. perfringens* CspC protein sequence alignments were performed with the Interactive Structure based Sequences Alignment Program (STRAP) using the ClustalW method. The locations of the catalytic residues for *C. perfringens* CspC, a subtilisin-like protease, were identified using the MEROPS database, which is maintained by the Wellcome Trust Sanger Institute. Catalytic residues (red), SNPs identified in the germination-null screen (green), SNP that alters germinant specificity (yellow).
(TIF)

Table S1 **Locations of the identified SNPs of the re-sequenced** ***C. difficile ger*** **isolates.** The locations of the identified SNPs of the re-sequenced *C. difficile* germination-null mutants are indicated. The 10 re-sequenced strains are color-coded in the table to aid in their identification. The position in the R20291 genome (Reference Position), the position in the re-sequenced UK1 genome (Consensus Position), the type of mutation (Variation Type), the number of nucleotides changed (Length), wild-type nucleotide sequence (Reference), the total number of variants (Variants), the identified SNP (Allele Variations), frequency of the identified SNPs at the given position (Frequencies), the number of reads that identified the SNP (Counts), the total number of reads at the position (Coverage), the called SNP (Variant #1), the frequency of the called SNP (Frequency of #1), the total number of reads of the called SNP (Count of #1), the annotation (Overlapping Annotations) and the impact of the mutation on the coding sequence (Amino Acid Change) are listed.
(XLSX)

Table S2 **Frequency of rifampin-resistant** ***C. difficile*** **UK1.** Exponential phase *C. difficile* cultures were exposed to EMS and then plated on agar medium or medium supplemented with rifampin. Wild-type, untreated *C. difficile* was included as a control.
(DOCX)

## References

[1] McDonald LC, Killgore GE, Thompson A, Owens RC Jr, Kazakova SV, et al (2005) An epidemic, toxin gene-variant strain of *Clostridium difficile* . N Engl J Med 353: 2433–2441. 16322603 10.1056/NEJMoa051590

[2] Redelings MD, Sorvillo F, Mascola L (2007) Increase in *Clostridium difficile*-related mortality rates, United States, 1999–2004. Emerging Infections Diseases 13: 1417–1419. 10.3201/eid1309.061116 PMC2857309 18252127

[3] Wilson KH, Perini F (1988) Role of competition for nutrients in suppression of *Clostridium difficile* by the colonic microflora. Infect Immun 56: 2610–2614. 3417352 10.1128/iai.56.10.2610-2614.1988 PMC259619

[4] Kuehne SA, Cartman ST, Heap JT, Kelly ML, Cockayne A, et al (2010) The role of toxin A and toxin B in *Clostridium difficile* infection. Nature 467: 711–713. 20844489 10.1038/nature09397

[5] Lyras D, O'Connor JR, Howarth PM, Sambol SP, Carter GP, et al (2009) Toxin B is essential for virulence of *Clostridium difficile* . Nature 458: 1176–9 doi:10.1038/nature07822. 19252482 10.1038/nature07822 PMC2679968

[6] Lawley TD, Croucher NJ, Yu L, Clare S, Sebaihia M, et al (2009) Proteomic and genomic characterization of highly infectious *Clostridium difficile* 630 spores. J Bacteriol 191: 5377–5386. 19542279 10.1128/JB.00597-09 PMC2725610

[7] Pruitt RN, Lacy DB (2012) Toward a structural understanding of *Clostridium difficile* toxins A and B. Front Cell Infect Microbiol 2: 28. 22919620 10.3389/fcimb.2012.00028 PMC3417631

[8] Carter GP, Rood JI, Lyras D (2012) The role of toxin A and toxin B in the virulence of *Clostridium difficile* . Trends Microbiol 20: 21–29. 22154163 10.1016/j.tim.2011.11.003

[9] Stragier P, Losick R (1996) Molecular Genetics of Sporulation in *Bacillus subtilis* . Annu Rev Genet 30: 297–341. 8982457 10.1146/annurev.genet.30.1.297

[10] Setlow P (2003) Spore germination. Curr Opin Microbiol 6: 550–556. 14662349 10.1016/j.mib.2003.10.001

[11] Paredes-Sabja D, Udompijitkul P, Sarker MR (2009) Inorganic phosphate and sodium ions are cogerminants for spores of *Clostridium perfringens* type A food poisoning-related isolates. Appl Environ Microbiol 75: 6299–6305. 19666724 10.1128/AEM.00822-09 PMC2753063

[12] Sebaihia M, Wren BW, Mullany P, Fairweather NF, Minton N, et al (2006) The multidrug-resistant human pathogen *Clostridium difficile* has a highly mobile, mosaic genome. Nat Genet 38: 779–786. 16804543 10.1038/ng1830

[13] Wilson KH (1983) Efficiency of various bile salt preparations for stimulation of *Clostridium difficile* spore germination. J Clin Microbiol 18: 1017–1019. 6630458 10.1128/jcm.18.4.1017-1019.1983 PMC270959

[14] Wilson KH, Kennedy MJ, Fekety FR (1982) Use of sodium taurocholate to enhance spore recovery on a medium selective for *Clostridium difficile* . J Clin Microbiol 15: 443–446. 7076817 10.1128/jcm.15.3.443-446.1982 PMC272115

[15] Railbaud P, Ducluzeau R, Muller MC, Sacquet E (1974) [Sodium taurocholate, a germination factor for anaerobic bacterial spores “in vitro” and “in vivo” (author's transl)]. Ann Microbiol (Paris) 125B: 381–391. 4282561

[16] Ridlon JM, Kang D, Hylemon PB (2006) Bile salt biotransformations by human intestinal bacteria. J Lipid Res 47: 241–259. 16299351 10.1194/jlr.R500013-JLR200

[17] Sorg JA, Sonenshein AL (2008) Bile salts and glycine as cogerminants for *Clostridium difficile* spores. J Bacteriol 190: 2505–2512. 18245298 10.1128/JB.01765-07 PMC2293200

[18] Sorg JA, Sonenshein AL (2009) Chenodeoxycholate is an inhibitor of *Clostridium difficile* spore germination. J Bacteriol 191: 1115–1117. 19060152 10.1128/JB.01260-08 PMC2632082

[19] Sorg JA, Sonenshein AL (2010) Inhibiting the initiation of *Clostridium difficile* spore germination using analogs of chenodeoxycholic acid, a bile acid. J Bacteriol 192: 4983–4990. 20675492 10.1128/JB.00610-10 PMC2944524

[20] Moir A, Lafferty E, Smith DA (1979) Genetic analysis of spore germination mutants of *Bacillus subtilis* 168: the correlation of phenotype with map location. J Gen Microbiol 124: 165–180. 10.1099/00221287-111-1-165 110906

[21] Kamiya S, Yamakawa K, Ogura H, Nakamura S (1989) Recovery of spores of *Clostridium difficile* altered by heat or alkali. J Med Microbiol 28: 217–221. 2926793 10.1099/00222615-28-3-217

[22] Masayama A, Hamasaki K, Urakami K, Shimamoto S, Kato S, et al (2006) Expression of germination-related enzymes, CspA, CspB, CspC, SleC, and SleM, of *Clostridium perfringens* S40 in the mother cell compartment of sporulating cells. Genes Genet Syst 81: 227–234. 17038794 10.1266/ggs.81.227

[23] Paredes-Sabja D, Setlow P, Sarker MR (2009) The protease CspB is essential for initiation of cortex hydrolysis and dipicolinic acid (DPA) release during germination of spores of *Clostridium perfringens* type A food poisoning isolates. Microbiology 155: 3464–3472. 19628563 10.1099/mic.0.030965-0

[24] Shimamoto S, Moriyama R, Sugimoto K, Miyata S, Makino S (2001) Partial characterization of an enzyme fraction with protease activity which converts the spore peptidoglycan hydrolase (SleC) precursor to an active enzyme during germination of *Clostridium perfringens* S40 spores and analysis of a gene cluster involved in the activity. J Bacteriol 183: 3742–3751. 11371539 10.1128/JB.183.12.3742-3751.2001 PMC95252

[25] Zhong J, Karberg M, Lambowitz AM (2003) Targeted and random bacterial gene disruption using a group II intron (targetron) vector containing a retrotransposition-activated selectable marker. Nucleic Acids Research 31: 1656–1664. 12626707 10.1093/nar/gkg248 PMC152852

[26] Heap JT, Pennington OJ, Cartmant ST, Carter GP, Minton NP (2007) The ClosTron: A universal gene knock-out system for the genus *Clostridium* . J Microbiol Methods 79: 452–464. 10.1016/j.mimet.2007.05.021 17658189

[27] Burns DA, Heap JT, Minton NP (2010) SleC is essential for germination of *Clostridium difficile* spores in nutrient-rich medium supplemented with the bile salt taurocholate. J Bacteriol 192: 657–664. 19933358 10.1128/JB.01209-09 PMC2812441

[28] Carter GP, Douce GR, Govind R, Howarth PM, Mackin KE, et al (2011) The anti-sigma factor TcdC modulates hypervirulence in an epidemic BI/NAP1/027 clinical isolate of *Clostridium difficile* . PLoS Pathog 7: e1002317. 22022270 10.1371/journal.ppat.1002317 PMC3192846

[29] Heeg D, Burns DA, Cartman ST, Minton NP (2012) Spores of *Clostridium difficile* clinical isolates display a diverse germination response to bile salts. PLoS One 7: e32381. 22384234 10.1371/journal.pone.0032381 PMC3285209

[30] Giel JL, Sorg JA, Sonenshein AL, Zhu J (2010) Metabolism of bile salts in mice influences spore germination in *Clostridium difficile* . PLoS One 5: e8740. 20090901 10.1371/journal.pone.0008740 PMC2806926

[31] Chang TW, Bartlett JG, Gorbach SL, Onderdonk AB (1978) Clindamycin-induced enterocolitis in hamsters as a model of pseudomembranous colitis in patients. Infect Immun 20: 526–529. 669810 10.1128/iai.20.2.526-529.1978 PMC421886

[32] Hudson KD, Corfe BM, Kemp EH, Feavers IM, Coote PJ, et al (2001) Localization of GerAA and GerAC germination proteins in the *Bacillus subtilis* spore. Journal of Bacteriology 183: 4317–4322. 11418573 10.1128/JB.183.14.4317-4322.2001 PMC95322

[33] Paidhungat M, Ragkousi K, Setlow P (2001) Genetic requirements for induction of germination of spores of *Bacillus subtilis* by Ca(2+)-dipicolinate. J Bacteriol 183: 4886–4893. 11466292 10.1128/JB.183.16.4886-4893.2001 PMC99543

[34] Adams CM, Eckenroth BE, Putnam EE, Doublie S, Shen A (2013) Structural and Functional Analysis of the CspB Protease Required for Clostridium Spore Germination. PLoS Pathog 9: e1003165. 23408892 10.1371/journal.ppat.1003165 PMC3567191

[35] Paredes-Sabja D, Sarker MR (2011) Germination response of spores of the pathogenic bacterium *Clostridium perfringens* and *Clostridium difficile* to cultured human epithelial cells. Anaerobe 17: 78–84. 21315167 10.1016/j.anaerobe.2011.02.001

[36] Howerton A, Patra M, Abel-Santos E (2013) A new strategy for the prevention of *Clostridium difficile* infections. J Infect Dis epub ahead of print. 10.1093/infdis/jit068 23420906

[37] Ramirez N, Liggins M, Abel-Santos E (2010) Kinetic evidence for the presence of putative germination receptors in *Clostridium difficile* spores. J Bacteriol 192: 4215–4222. 20562307 10.1128/JB.00488-10 PMC2916422

[38] Howerton A, Ramirez N, Abel-Santos E (2010) Mapping interactions between germinants and *C. difficile* spores. J Bacteriol 193: 274–282. 20971909 10.1128/JB.00980-10 PMC3019946

[39] Allen CA, Babakhani F, Sears P, Nguyen L, Sorg JA (2013) Both Fidaxomicin and Vancomycin Inhibit Outgrowth of *Clostridium difficile* Spores. Antimicrob Agents Chemother 57: 664–667. 23147724 10.1128/AAC.01611-12 PMC3535933

[40] Hanahan D (1983) Studies on transformation of *Escherichia coli* with plasmids. J Mol Biol 166: 557–580. 6345791 10.1016/s0022-2836(83)80284-8

[41] Bouillaut L, McBride SM, Sorg JA (2011) Genetic manipulation of *Clostridium difficile* . Curr Protoc Microbiol Chapter 9: Unit 9A 2. 10.1002/9780471729259.mc09a02s20 PMC3615975 21400677

[42] Wren BW, Tabaqchali S (1987) Restriction endonuclease DNA analysis of *Clostridium difficile* . J Clin Microbiol 25: 2402–2404. 2828418 10.1128/jcm.25.12.2402-2404.1987 PMC269500

[43] Bouillaut L, Self WT, Sonenshein AL (2012) Proline-Dependent Regulation of *Clostridium difficile* Stickland Metabolism. J Bacteriol epub ahead of print. 10.1128/JB.01492-12 PMC3562115 23222730

[44] Heap JT, Pennington OJ, Cartman ST, Minton NP (2009) A modular system for Clostridium shuttle plasmids. J Microbiol Methods 78: 79–85. 19445976 10.1016/j.mimet.2009.05.004

[45] Permpoonpattana P, Tolls EH, Nadem R, Tan S, Brisson A, et al (2011) Surface layers of *Clostridium difficile* endospores. J Bacteriol 193: 6461–6470. 21949071 10.1128/JB.05182-11 PMC3232898

[46] Cabrera-Martinez R-M, Tovar-Rojo F, Vepachedu VR, Setlow P (2003) Effects of overexpression of nutrient receptors on germination of spores of *Bacillus subtilis* . J Bacteriol 185: 2457–2464. 12670969 10.1128/JB.185.8.2457-2464.2003 PMC152624

[47] Sambol SP, Tang JK, Merrigan MM, Johnson S, Gerding DN (2001) Infection of hamsters with epidemiologically important strains of *Clostridium difficile* . J Infect Dis 183: 1760–1766. 11372028 10.1086/320736

[48] Haraldsen JD, Sonenshein AL (2003) Efficient sporulation in *Clostridium difficile* requires disruption of the sigmaK gene. Molecular Microbiology 48: 811–821. 12694623 10.1046/j.1365-2958.2003.03471.x

