# Supplementary figures and images for: Bile Acid Recognition by the Clostridium difficile Germinant Receptor, CspC, Is Important for Establishing Infection
Source: PLoS Pathog. 2013 May 9;9(5):e1003356. doi: 10.1371/journal.ppat.1003356 (PMC3649964; doi:10.1371/journal.ppat.1003356)

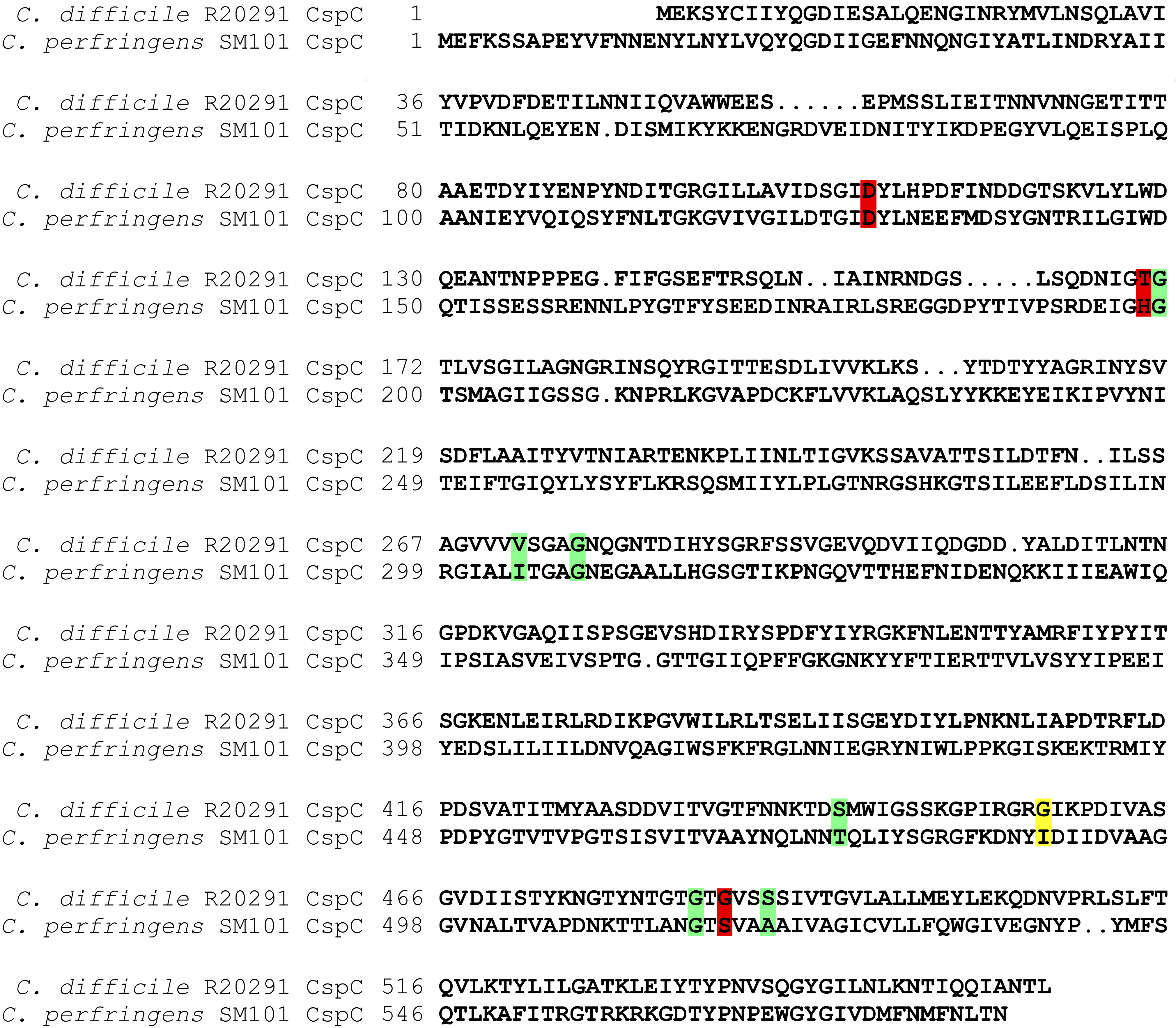

Supplement: Figure S1 — Sequence alignment between C. difficile CspC and C. perfringens CspC. C. difficile CspC and C. perfringens CspC protein sequence alignments were performed with the Interactive Structure based Sequences Alignment Program (STRAP) using the ClustalW method. The locations of the catalytic residues for C. perfringens CspC, a subtilisin-like protease, were identified using the MEROPS database, which is maintained by the Wellcome Trust Sanger Institute. Catalytic residues (red), SNPs identified in the germination-null screen (green), SNP that alters germinant specificity (yellow). (TIF) [file ppat.1003356.s001.tif]
