# Supplementary material for: Bile Acid Recognition by the Clostridium difficile Germinant Receptor, CspC, Is Important for Establishing Infection
Source: PLoS Pathog. 2013 May 9;9(5):e1003356. doi: 10.1371/journal.ppat.1003356 (PMC3649964; doi:10.1371/journal.ppat.1003356)
Supplement: Table S2 — Frequency of rifampin-resistant C. difficile UK1. Exponential phase C. difficile cultures were exposed to EMS and then plated on agar medium or medium supplemented with rifampin. Wild-type, untreated C. difficile was included as a control. (DOCX) [file ppat.1003356.s003.docx]

Table S2. Frequency of rifampin-resistant *C. difficile* UK1

|  | *C. difficile* UK1 | *C. difficile* + 1% EMS |
| --- | --- | --- |
| Total Cells | 2.8 x 10^8^ | 4.3 x 10^7^ |
| Total Rif^R^ Cells | 10 | 1.6 x 10^3^ |
| Frequency | 3.6 x 10^-8^ | 3.7 x 10^-5^ |
